# CNV-ClinViewer: enhancing the clinical interpretation of large copy-number variants online

**DOI:** 10.1093/bioinformatics/btad290

**Published:** 2023-04-27

**Authors:** Marie Macnee, Eduardo Pérez-Palma, Tobias Brünger, Chiara Klöckner, Konrad Platzer, Arthur Stefanski, Ludovica Montanucci, Allan Bayat, Maximilian Radtke, Ryan L Collins, Michael Talkowski, Daniel Blankenberg, Rikke S Møller, Johannes R Lemke, Michael Nothnagel, Patrick May, Dennis Lal

**Affiliations:** Cologne Center for Genomics (CCG), University of Cologne, Cologne, Germany; Universidad del Desarrollo, Centro de Genética y Genómica, Facultad de Medicina Clínica Alemana, Santiago, Chile; Cologne Center for Genomics (CCG), University of Cologne, Cologne, Germany; Institute of Human Genetics, University of Leipzig Medical Center, Leipzig, Germany; Institute of Human Genetics, University of Leipzig Medical Center, Leipzig, Germany; Genomic Medicine Institute, Lerner Research Institute, Cleveland Clinic, Cleveland, OH, USA; Epilepsy Center, Neurological Institute, Cleveland Clinic, Cleveland, OH, USA; Genomic Medicine Institute, Lerner Research Institute, Cleveland Clinic, Cleveland, OH, USA; Department of Epilepsy Genetics and Personalized Medicine, Member of ERN Epicare, Danish Epilepsy Centre, Dianalund, Denmark; Department of Regional Health Research, Faculty of Health Sciences, University of Southern Denmark, Odense, Denmark; Institute of Human Genetics, University of Leipzig Medical Center, Leipzig, Germany; Broad Institute of Massachusetts Institute of Technology and Harvard, Cambridge, MA, USA; Center for Genomic Medicine, Massachusetts General Hospital, Harvard Medical School, Boston, MA, USA; Broad Institute of Massachusetts Institute of Technology and Harvard, Cambridge, MA, USA; Center for Genomic Medicine, Massachusetts General Hospital, Harvard Medical School, Boston, MA, USA; Genomic Medicine Institute, Lerner Research Institute, Cleveland Clinic, Cleveland, OH, USA; Department of Epilepsy Genetics and Personalized Medicine, Member of ERN Epicare, Danish Epilepsy Centre, Dianalund, Denmark; Department of Regional Health Research, Faculty of Health Sciences, University of Southern Denmark, Odense, Denmark; Institute of Human Genetics, University of Leipzig Medical Center, Leipzig, Germany; Cologne Center for Genomics (CCG), University of Cologne, Cologne, Germany; University Hospital Cologne, Cologne, Germany; Luxembourg Centre for Systems Biomedicine, University Luxembourg, Esch-sur-Alzette, Luxembourg; Cologne Center for Genomics (CCG), University of Cologne, Cologne, Germany; Genomic Medicine Institute, Lerner Research Institute, Cleveland Clinic, Cleveland, OH, USA; Epilepsy Center, Neurological Institute, Cleveland Clinic, Cleveland, OH, USA; Broad Institute of Massachusetts Institute of Technology and Harvard, Cambridge, MA, USA

## Abstract

**Motivation:**

Pathogenic copy-number variants (CNVs) can cause a heterogeneous spectrum of rare and severe disorders. However, most CNVs are benign and are part of natural variation in human genomes. CNV pathogenicity classification, genotype–phenotype analyses, and therapeutic target identification are challenging and time-consuming tasks that require the integration and analysis of information from multiple scattered sources by experts.

**Results:**

Here, we introduce the CNV-ClinViewer, an open-source web application for clinical evaluation and visual exploration of CNVs. The application enables real-time interactive exploration of large CNV datasets in a user-friendly designed interface and facilitates semi-automated clinical CNV interpretation following the ACMG guidelines by integrating the ClassifCNV tool. In combination with clinical judgment, the application enables clinicians and researchers to formulate novel hypotheses and guide their decision-making process. Subsequently, the CNV-ClinViewer enhances for clinical investigators’ patient care and for basic scientists’ translational genomic research.

**Availability and implementation:**

The web application is freely available at https://cnv-ClinViewer.broadinstitute.org and the open-source code can be found at https://github.com/LalResearchGroup/CNV-clinviewer.

## 1 Introduction

One of the reasons for genetic disorders is copy-number changes of one or more genes, resulting from deletions, duplications, or other genomic rearrangements. Most disease-associated CNVs are unique, cause severe complex disorders, and are often hard to distinguish from benign CNVs frequently found in the general population (Riggs et al. 2020).

Although the average size of pathogenic/likely pathogenic CNVs in ClinVar (Landrum et al. 2018) of 9.7 Mb (95% CI 9.3–10.1 Mb, median = 2.6 Mb) is likely overestimated due to frequent imprecise discovery of CNVs with arrays, disease-associated CNVs typically cover tens to hundreds of genes. Thus, pinpointing the underlying disease driver and modifier genes represents a major challenge. Given the complexity of CNV interpretation, the American College of Medical Genetics and Genomics (ACMG) has developed technical standards (a quantitative, evidence-based scoring framework) to standardize the evaluation process of the genomic content of a CNV region, and to promote consistency and transparency in classification and reporting across clinical laboratories (Riggs et al. 2020). The application of those standards involves clinical and genetic CNV data that are scattered across registries, databases, and the literature. Therefore, it is difficult and highly time-consuming to annotate, analyze, and interpret CNVs manually in a clinical setting.

Several bioinformatics tools, often fully-automated, have been developed for clinicians and researchers to facilitate large-scale CNV classification according to the ACMG guidelines (for example see (Geoffroy et al. 2018, Gurbich and Ilinsky 2020, Requena et al. 2021). However, no algorithm or even classification framework can perfectly capture data, i.e. usually only available to experts, such as deep clinical, CNV, or gene-level information. This specifically affects data that cannot be extracted from public online resources, such as detailed information about the phenotype, including family history, CNV inheritance pattern, and many other criteria currently not integrated in the clinical significance classification. For the semi-automated tools (Fan et al. 2021), the extracted and manually entered information is not explorable within the same web-application interface and comparative visual inspection of CNVs with various genomic and clinical data sources is not possible because current tools do not provide such functionality (Geoffroy et al. 2018, Gurbich and Ilinsky 2020, Fan et al. 2021, Requena et al. 2021). While the current focus of those existing tools is on clinical significance classification, they are not designed to perform ad-hoc evaluations of new data such as genotype–phenotype analyses to expose undiscovered patterns of CNV localization in patient cohorts. To show a correlation of patient CNVs with known disease genes at a specific locus or to narrow down dosage-sensitive genes that are likely driver genes in particular CNVs, the interactive visual inspection of affected genomic content combined with crosslinked data from various sources can enhance the interpretational analysis of CNVs. Paradoxically, the individuals who collect the most relevant clinical information for CNV clinical significance classification and interpretation, such as genetic counselors, treating physicians, and patients’ families, often lack the required bioinformatical expertise to examine several patient CNVs simultaneously and integrate information from multiple scattered sources time-efficiently. To overcome current limitations for biomedical CNV interpretation of those individuals and researchers, we developed the CNV-ClinViewer, a fully open-source exploration and interpretation platform that integrates analysis, annotation, guideline based, classification and clinical evaluation of CNVs and provides users, even families with CNVs of uncertain significance, with an interface to curate, visualize, interact with, and continuously re-evaluate the CNV data.

## 2 Materials and methods

Details about the data sources used in the CNV-ClinViewer can be found in Supplementary Table S1.

### 2.1 Genetic CNV variants and regions datasets

CNVs with annotated clinical significance were obtained from ClinVar (Landrum et al. 2018) (updated quarterly), and CNVs identified in the general population were obtained from the UK biobank (Aguirre et al. 2019) as well as from gnomAD (Collins et al. 2020) (controls-only dataset, version 2.1). GnomAD CNVs were reduced to those with a filter equal to PASS, and SVTYPE equivalent to “DEL” or “DUP”, and all CNVs from the general population were filtered for large CNVs >50 kb. Specifically, we collected 52 344 CNVs from ClinVar (10 654 pathogenic/likely pathogenic CNVs), 195 916 CNVs from the UK-biobank (>50 kb), and 10 370 CNVs from gnomAD (>50 kb). The genome coordinates of CNVs from the UK biobank and gnomAD were converted to GRCh38 using the UCSC LiftOver tool (Kent et al. 2002, Riggs et al. 2020).

To annotate neutral as well as clinically relevant regions by use of a visual summary of the variants, we further processed the variants by counting the number of deletions or duplications in the different datasets using a sliding window approach with a window size of 200 kb and step size of 100 kb. For the cohort-level data from the UK-biobank and gnomAD, we divided the counts by the number of samples in the cohort to retrieve region-specific allele frequencies.

Known CNV syndromes and genomic regions with evaluated dosage sensitivity were obtained from DECIPHER (Firth et al. 2009) and ClinGen (Rehm et al. 2015) (updated quarterly) databases.

### 2.2 Gene-level annotations

We retrieved a list of 18 792 protein-coding gene symbols from the HUGO Gene Nomenclature Committee (HGNC) (Tweedie et al. 2021). These genes were annotated by genomic boundaries of one transcript from RefSeq (O’Leary et al. 2016) (GRCh37/hg19 and GRCh38/hg38), either the MANE select transcript or alternatively the longest transcript. In addition, we annotated gene-level scores for sequence constraint and dosage sensitivity in humans (Table 1) and gene–disease associations from ClinGen (Rehm et al. 2015) (updated quarterly).

**Table 1.** Description of gene-level scores for sequence constraint and dosage sensitivity^a^

Category	Score	Description	Threshold
Sequence constraint score as proxy for haploinsufficiency	pLI (Lek et al. 2016)	Probability of loss of function intolerance. Genes with high pLI scores are considered more intolerant to protein truncation variations.	>0.9
Sequence constraint score as proxy for haploinsufficiency	loeuf (Karczewski et al. 2020)	Loss-of-function observed/expected upper bound fraction. Low LOEUF scores indicate strong selection against predicted loss-of-function (pLoF) variation in a given gene.	<0.35
Haploinsufficiency	pHI (Collins et al. 2022)	Predicted probability of haploinsufficiency of autosomal genes by predictive model from a cross-disorder dosage sensitivity map of the human genome (analysis of rare CNVs from >700k individuals). Genes with high pHI scores are considered more likely triplosensitive.	>0.833
Triplosensitivity	pTS (Collins et al. 2022)	Predicted probability of triplsensitivity of autosomal genes by predictive model from a cross-disorder dosage sensitivity map of the human genome (analysis of rare CNVs from >700k individuals). Genes with high pTS scores are considered more likely triplosensitive.	>0.993
Haploinsufficiency	%HI (Firth et al. 2009)	By DECIPHER (Firth et al. 2009) updated predictions of haploinsufficiency as described by (Huang et al. (2010). Genes with high ranks (e.g. 0–10%) indicate a gene is more likely to exhibit haploinsufficiency.	<10%
Haploinsufficiency	HI Score ClinGen (Rehm et al. 2015)	The ClinGen Dosage Sensitivity expert group collects evidence supporting/refuting the haploinsufficiency of genes.	3 (sufficient evidence)
Triplosensitivity	TS Score ClinGen (Rehm et al. 2015)	The ClinGen Dosage Sensitivity expert group collects evidence supporting/refuting the triplosensitivity of genes.	3 (sufficient evidence)

a The given thresholds are used in the CNV-ClinViewer to annotate dosage sensitivity of genes.

### 2.3 CNV-ClinViewer web server development

The CNV-ClinViewer was developed with the Shiny framework of R studio software (v.1.7.1, https://shiny.rstudio.com/) which transforms regular R code into an interactive environment that can follow and “react” to remote-user instructions. The pre-processed data alongside the R/Shiny code was uploaded as a stand-alone Ubuntu image with Google Cloud services. The image was deployed into a Google Virtual Machine (VM) using the googleComputeEngineR package (v.0.3.0, https://github.com/cloudyr/googleComputeEngineR). The CNV-ClinViewer web server (https://cnv-ClinViewer.broadinstitute.org/) is compatible with all commonly used Internet browsers.

For the classification of uploaded CNVs, the clinical interpretation tool ClassifyCNV (Gurbich and Ilinsky 2020) including its own data sources was integrated into the CNV-ClinViewer framework. Upon selection of one of the uploaded and classified CNVs, all pre-processed data are intersected by genomic coordinates of the selected CNV. Consequently, the HTML report describing the selected CNV is generated using the rmarkdown R package (v2.11, https://rmarkdown.rstudio.com), and the interactive visualizations and tables are rendered using the ggplot2 (v.3.3.5, https://ggplot2.tidyverse.org), plotly (v.4.9.4.1 https://plotly-r.com), and DT (v.0.19, https://CRAN.R-project.org/package=DT) R packages. For the gene set enrichment analysis, the enrichr R package (v3.0, https://CRAN.R-project.org/package=enrichR) that provides an interface to the Enrichr database is used.

The CNV-ClinViewer is an open-source project, and its code will continue to grow and improve through version control in the GitHub repository (https://github.com/LalResearchGroup/CNV-clinviewer).

## 3 Results

**Figure 1.**
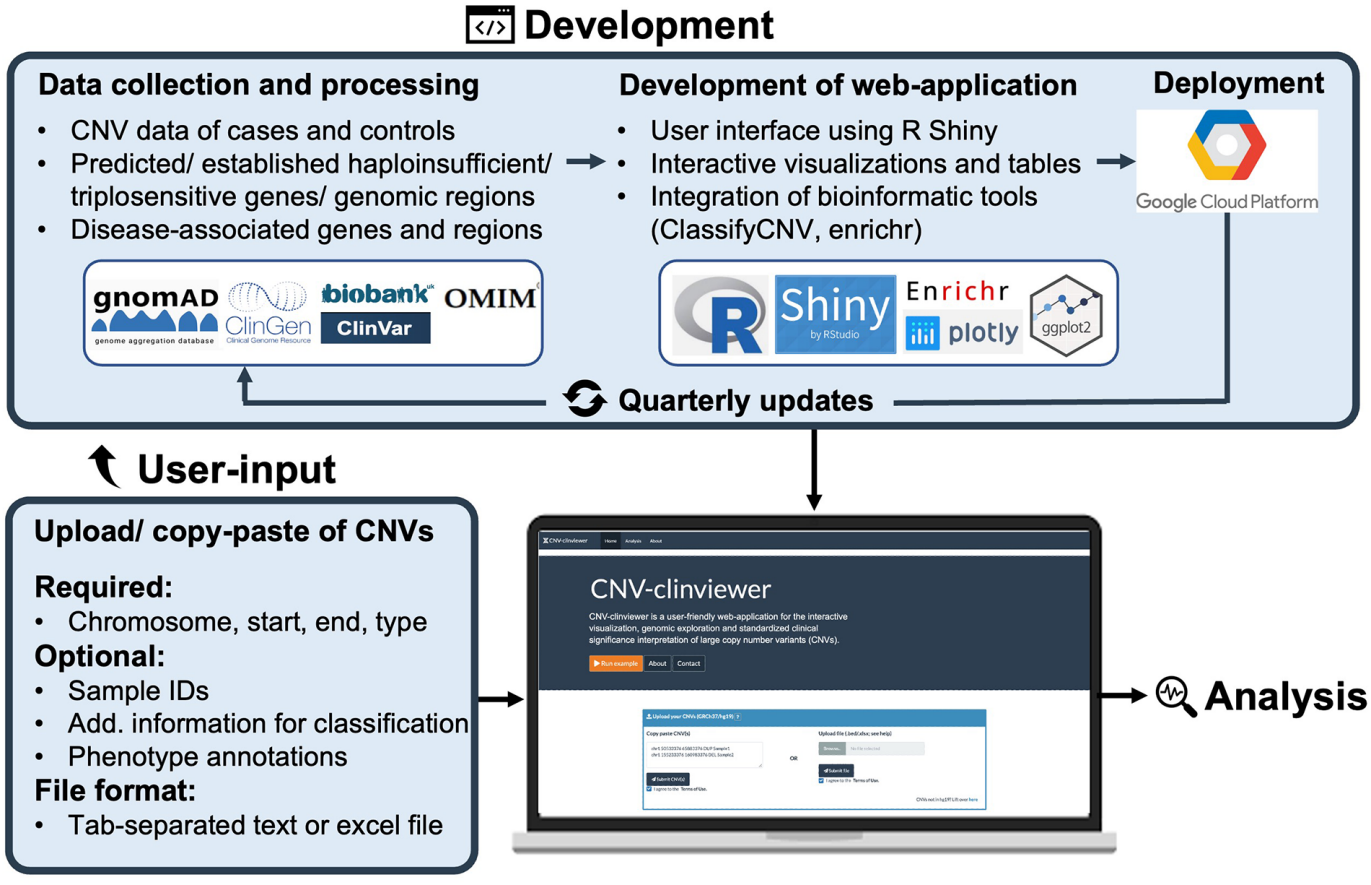
Development and use of CNV-ClinViewer. CNV data as well as clinical and genomic annotations are collected, processed and annotated, and quarterly updated. Users can copy-paste CNV(s) or upload CNVs in a tab-separated or Excel file for real-time exploration and interpretation.

We present the CNV-ClinViewer, a user-friendly web application that semi-automatedly enhances the ACMG guideline-based CNV classification (Kuleshov et al. 2016) for caregivers and facilitates the biomedical interpretation of gene content at a locus of interest for scientists. Development, workflow, and main features are presented in Fig. 1.

### 3.1 Upload of one up to thousands of CNVs for clinical interpretation and genetic reports

The CNV-ClinViewer allows simultaneous analysis of single or multiple CNVs, irrespective of the technology used to identify them. It requires as input the genomic coordinates of CNVs based on the human reference genome GRCh37/hg19 (https://www.ncbi.nlm.nih.gov/data-hub/genome/GCF_000001405.13/) or GRCh38/hg38 (https://www.ncbi.nlm.nih.gov/data-hub/genome/GCF_000001405.40/), either by copy-pasting or by uploading a tab-separated text or Excel file (max. number of CNVs = 10 000). Minimal required information for each CNV, including whole chromosome trisomies and monosomies, is the chromosome, start, end, and CNV type (deletion or duplication). Optionally, the user can provide sample IDs, phenotype information for filtering and a score of manually assessed evidence categories of the 2019 ACMG standard guidelines (see below). Details and example files can be found in the help section and on the about page of the CNV-ClinViewer.

**Figure 2.**
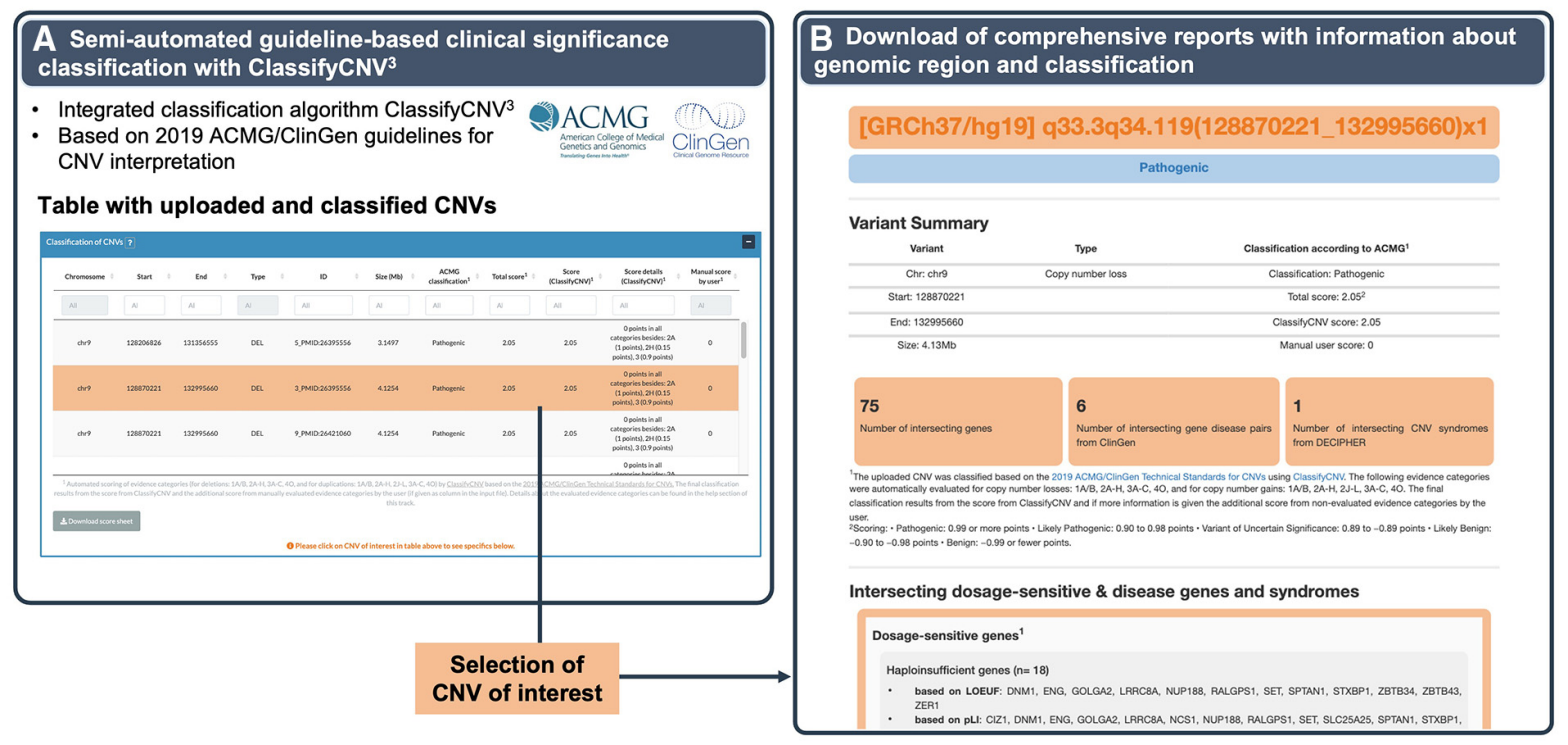
Semi-automated classification and download of CNV reports. (**A**) The integrated semi-automated classification of uploaded CNVs is based on 2019 ACMG/ClinGen Technical Standards for CNVs by ClassifyCNV^3^ and is one of the key features of the CNV-ClinViewer. The resulting scores and applied evidence categories can be inspected in a table overview and also downloaded. (**B**) A comprehensive report on individual CNVs, including details about the clinical significance classification and the overlap with established/predicted haploinsufficient/triplosensitive and clinically relevant genes and genomic regions, can be downloaded.

After CNV submission, the user is directed to the analysis interface. Here, five different analysis panels are available. In the *first analysis panel*, the uploaded CNVs are displayed in a downloadable table comprising their annotated clinical significance and details about the scoring (Fig. 2A). The CNVs are classified by ClassifyCNV (Gurbich and Ilinsky 2020), a command-line tool integrated into the CNV-ClinViewer infrastructure that automatically evaluates the evidence categories 1A/B, 2A–H, 3A–C, 4O for copy-number losses and 1A/B, 2A–H, 2J–L, 3A–C, 4O for copy-number gains from the 2019 ACMG/ClinGen Technical Standards for CNVs (Riggs et al. 2020). In case the user uploaded additional scores of relevant ACMG clinical significance criteria for which additional information, such as family history or the *de novo* status, is required, the score is added to the score from ClassifyCNV to refine the final score and classification. In the *second analysis panel*, a comprehensive report of the individual CNVs can be downloaded (Fig. 2B). The report includes details about the clinical significance classification and the overlap with established/predicted haploinsufficient/triplosensitive and clinically relevant genes and genomic regions.

### 3.2 Uploaded CNV data can be visually inspected alongside publicly available data

**Figure 3.**
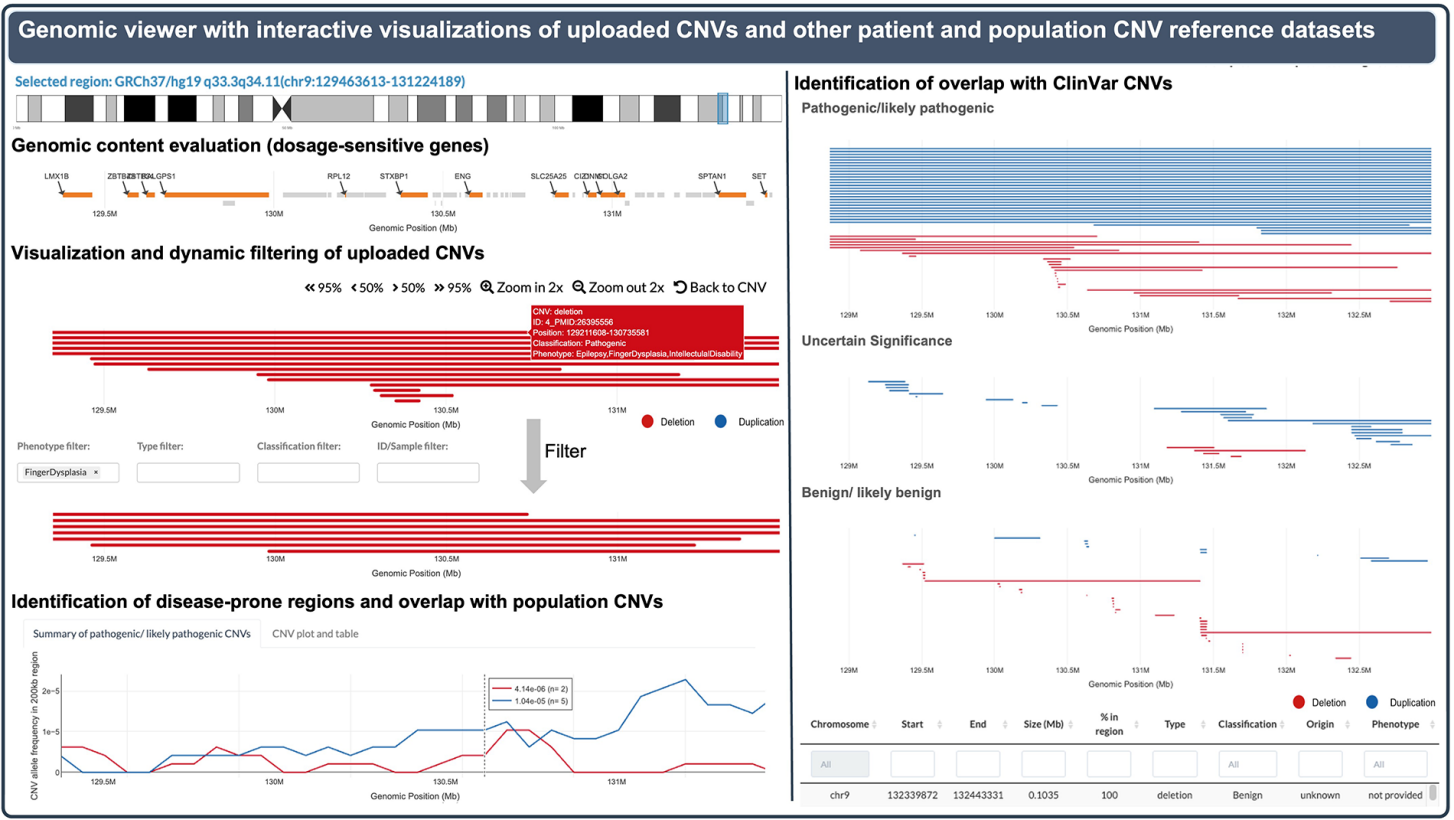
Genomic viewer. A genomic viewer allows inspecting the uploaded CNVs and their genomic region alongside biomedical annotations and other pathogenic and general population CNV datasets. Here, the user can visually compare and dynamically filter uploaded CNVs, perform a seamless evaluation of the gene content, identify disease-prone regions and find unknown patterns of CNV localization to generate hypotheses for further research. An extended view of the genomic viewer can be found in Supplementary Fig. S1.

The *third panel* of the analysis interface is a genomic viewer (Fig. 3 and Supplementary Fig. S1A–F). Here, the uploaded CNVs and their genomic region can be inspected alongside biomedical annotations and other pathogenic and general population CNV datasets. Included are five data tracks that the user can interactively navigate by zooming in/out, moving horizontally, and selecting genomic regions of interest. In the first track of the genomic viewer (Supplementary Fig. S1B), the CNV-ClinViewer integrates seamless evaluation of the gene content by visualization of all protein-coding genes. Here, the genes are highlighted by a selection of gene dosage sensitivity scores (Table 1) to enable fast and visual gene prioritization in context to the uploaded CNVs. Below this gene track, all uploaded CNVs that intersect the selected region are visualized and can be interactively filtered based on uploaded phenotypic annotations, their assigned clinical significance, CNV type, and sample IDs (Supplementary Fig. S1C). The further tracks enable the visual identification of disease-prone regions with coupled visualizations of summarized data (allele frequencies and allele counts) of more than 250 000 CNVs from the general population [UK Biobank (Aguirre et al. 2019), gnomAD (Collins et al. 2020)], and pathogenic/likely pathogenic CNVs from ClinVar (Landrum et al. 2018) (Supplementary Fig. S1D–F). In addition, users can identify the overlap of uploaded CNVs with individual ClinVar CNVs (Supplementary Fig. S1D). The latter are displayed in an interactive plot grouped by their assigned clinical significance (pathogenic/likely pathogenic versus uncertain significance versus benign/likely benign) and can be filtered based on their type and clinical significance. Details about the ClinVar CNVs, such as their reported phenotypes or allele origin and the link to the ClinVar variant website, are shown in a table and can be downloaded for further analyses.

### 3.3 Additional features and analyses provide users with advanced CNV insights

**Figure 4.**
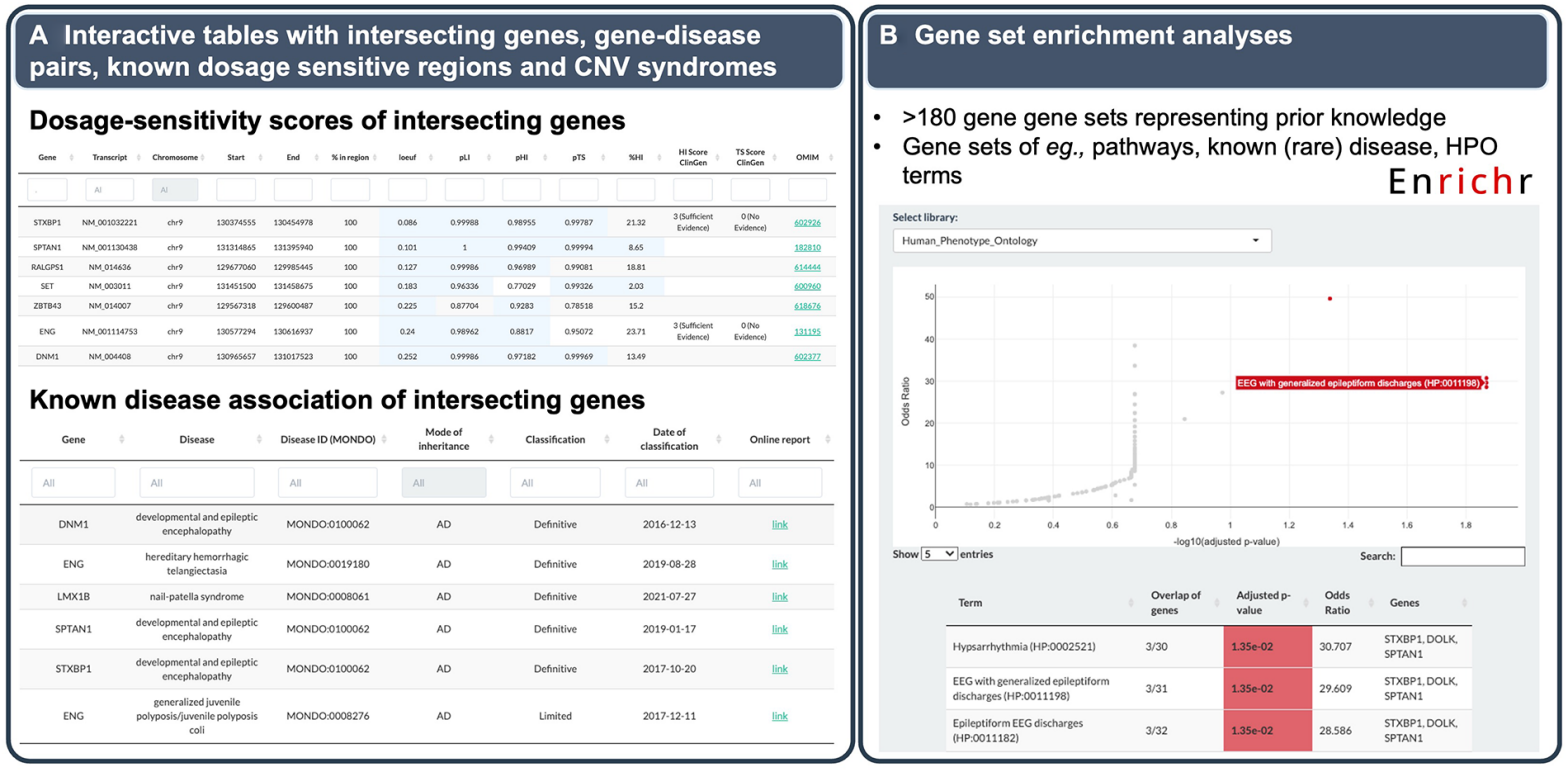
Additional features. (**A**) Users can retrieve, filter, and download information about the intersecting genes, gene–disease associations, known dosage sensitive regions, and known CNV syndromes. (**B**) Users can perform gene-set enrichment analyses (GSEA) to infer information on genes within a selected genomic region or CNV by comparing it to >180 annotated gene sets representing prior biological knowledge such as pathways, Human Phenotype Ontology (HPO) terms, and known (rare) diseases.

Below the genomic viewer, the user can retrieve and download information about the intersecting genes, classified gene–disease associations from ClinGen (Rehm et al. 2015), known dosage-sensitive regions and CNV syndromes (Fig. 4A). The gene table contains all protein-coding genes, their genomic coordinates and transcript IDs, links to the Online Mendelian Inheritance in Man database (OMIM, https://omim.org/), and gene dosage sensitivity scores (Table 1). All genes classified by ClinGen with reported associations with one or several diseases are shown in the gene–disease association table, including links to the comprehensive gene–disease association reports on the ClinGen website. In the ClinGen region table and the DECIPHER (Firth et al. 2009) CNV syndrome table, all well-characterized dosage sensitive regions and developmental CNV syndromes that intersect a CNV/genomic region of interest can be retrieved.

In the last panel, the user can perform gene set enrichment analyses to infer knowledge about the genes from a selected genomic region by comparing it to more than 180 annotated gene sets representing prior biological knowledge such as pathways, Human Phenotype Ontology (HPO) terms (Köhler et al. 2021), and known (rare) diseases (Fig. 4B).

### 3.4 Example analysis: 9q33.3q34.11 microdeletions

To illustrate the utility of the CNV-ClinViewer, we performed an example analysis of 14 previously published CNVs (9q33.3q34.11 microdeletions) from patients with complex developmental disorders from the literature (*n* = 14) (Campbell et al. 2012, Saitsu et al. 2012, Ehret et al. 2015, Nambot et al. 2016). Here, the CNVs were first uploaded and automatically classified as (likely) pathogenic (Supplementary Figs S2 and S3). Next, the smallest region of overlap with its dosage sensitive gene *STXBP1* could be immediately identified due to the interactive visualization of the overlapping patient CNVs (Supplementary Figs S4 and S5). In addition, the CNV-ClinViewer could resolve the clinical heterogeneity of patients by enabling the user to filter the CNVs based on assigned phenotypes (Supplementary Fig. S7) and retrieve region-specific information about intersecting clinically relevant genes and their associated phenotypes (Supplementary Fig. S6) as well as overlapping patient CNVs from ClinVar (Supplementary Fig. S8). Overall, we demonstrate the CNV-ClinViewer’s fast and seamless biomedical interpretation of CNVs and its potential to replicate research findings due to a large number of annotations and visual inspection capabilities. To perform a comparable analysis without the CNV-ClinViewer, the user would be required to classify the CNVs manually one at a time or with the support of an existing tool, visualize the CNVs with yet another tool or genomic viewer, visit several webpages, and process and annotate information from various databases. The example analysis, including an example of benign CNVs of chromosome 9, can be found in more detail and with step-by-step instructions illustrated with images in the Supplementary File.

## 4 Discussion

By aggregating more than 250 000 population and patient CNVs, annotating various gene scores and clinical annotations, and integrating useful existing bioinformatics tools, we developed a novel and user-friendly interface as a decision support tool for the clinical evaluation of CNVs and comparative visual inspection.

CNV analysis and interpretation tools and, in general, clinical decision support tools can make patient care more efficient, cost-effective, and guideline concordant. However, although the prediction results from many approaches are promising, their value is limited by their lack of interpretability and human intuition. The black-box nature of some pathogenicity predictions can further exacerbate trust issues and worsen the overall experience (Cai et al. 2019). To address this issue, human-centered tools with interactive mechanisms can grant end-users more agency in guiding the interpretation and can be used for critical decision-making purposes beyond an algorithm (Cai et al. 2019, Babione et al. 2020). Therefore, in the development of the CNV-ClinViewer, we emphasized interactive analyses of the CNVs beyond a classification score and allow the user to dynamically inspect and compare the results in detail. In comparison, existing open-source tools (Geoffroy et al. 2018, Gurbich and Ilinsky 2020, Fan et al. 2021) focus more on the classification scores than on the interactive inspection and further investigations. We believe that the comprehensive approach of the CNV-ClinViewer will empower users with more trust in the results, the agency to test hypotheses, and enable them to apply their domain knowledge while simultaneously leveraging the benefits of automation.

To ensure that a tool is easy to use and is designed for target users at each development stage, human-centered design methods and principles should be applied (Babione et al. 2020, Bates et al. 2003). Such methods draw from well-established design principles in many disciplines, including usability, visualizations, and interface design, and consider users’ context and experience. As the effectiveness of clinical tools may be severely limited without the application of those principles and could even contribute to adverse medical events (Graham et al. 2008), we put great emphasis on developing a user-friendly interface with simple data upload with minimal requirements, a clear analysis workflow, and real-time results, while giving the user the ability to retrieve details and help and modify the analysis when required. We also focused on the visual and intuitive inspection of CNV data and its interaction with the user. In particular, for genomic data, the integration of visualizations is essential for interpretation and hypothesis generation, essential for combining different data sources and discovering unexpected localization patterns of genomic annotations in a short amount of time, as well as a valuable aid in communicating discoveries (Nusrat et al. 2019).

On the one hand, the CNV-ClinViewer will assist clinicians, clinical geneticists, and genetic counselors in analyzing and interpreting CNV data and improving objectivity and accuracy in the clinical work-up. On the other hand, the collected and integrated knowledge combined with clinical judgment will enable clinicians and researchers to formulate novel hypotheses and guide their decision-making process.

The current features of the tool are focused on large CNVs that intersect several genes when gene prioritization plays a role in the analysis. Future versions will increase the granularity of the tool, e.g. by providing a more detailed gene track that includes exons and introns, and different transcripts with expression data. With that, it will bring more value for interpreting intragenic deletions and duplications. Also, to integrate with existing workflows, we plan to develop an application programming interface (API) to query the system and fetch the results without using a graphical user interface. Overall, the CNV-ClinViewer is the first framework that enables interactive exploration of CNV data and semi-automated variant classification. With its scalable architecture, the web application is well suited for both genome-wide (re-)evaluations of large datasets and the small number of CNVs. The CNV-ClinViewer is an open-source tool (https://www.cnv-ClinViewer.broadinstitute.org/), including complete documentation on the use of the website.

## Supplementary Material

btad290_Supplementary_Data

## Data Availability

The data used in the CNV-ClinViewer are listed in Supplementary Table S1. All code and data are accessible at https://github.com/LalResearchGroup/CNV-clinviewer, and detailed documentation can be found at https://www.cnv-ClinViewer.broadinstitute.org/. The web application is free and open to all users, and there is no login requirement.
