# Supplementary material for: CNV-ClinViewer: enhancing the clinical interpretation of large copy-number variants online
Source: Bioinformatics. 2023 Apr 27;39(5):btad290. doi: 10.1093/bioinformatics/btad290 (PMC10174702; doi:10.1093/bioinformatics/btad290)
Supplement: btad290_Supplementary_Data [file btad290_supplementary_data.docx]

Supplementary information file

**Title: CNV-ClinViewer: Enhancing the clinical interpretation of large copy-number variants online**

Marie Macnee^1^, Eduardo Pérez-Palma^2^, Tobias Brünger^1^, Chiara Klöckner^3^, Konrad Platzer^3^, Arthur Stefanski^4-5^, Ludovica Montanucci^4^, Allan Bayat^6-7^, Maximilian Radtke^3^, Ryan Collins^8-9^, Michael Talkowski^8-9^, Daniel Blankenberg^4^, Rikke S Møller^6-7^, Johannes R Lemke^3^, Michael Nothnagel^1,10^, Patrick May^11^, Dennis Lal^1,4-5,8^

^1^Cologne Center for Genomics (CCG), University of Cologne, Cologne, Germany.

^2^Universidad del Desarrollo, Centro de Genética y Genómica, Facultad de Medicina Clínica Alemana, Santiago, Chile.

^3^Institute of Human Genetics, University of Leipzig Medical Center, Leipzig, Germany.

^4^Genomic Medicine Institute, Lerner Research Institute, Cleveland Clinic, Cleveland, OH, USA.

^5^Epilepsy Center, Neurological Institute, Cleveland Clinic, Cleveland, OH, USA.

^6^Department of Epilepsy Genetics and Personalized Medicine, Member of ERN Epicare, Danish Epilepsy Centre, Dianalund, Denmark.

^7^Department of Regional Health Research, Faculty of Health Sciences, University of Southern Denmark, Denmark.

^8^Broad Institute of Massachusetts Institute of Technology and Harvard, Cambridge, MA, USA.

^9^Center for Genomic Medicine, Massachusetts General Hospital, Harvard Medical School, Boston, MA, USA.

^10^University Hospital Cologne, Cologne, Germany.

^11^Luxembourg Centre for Systems Biomedicine, University Luxembourg, Esch-sur-Alzette, Luxembourg.

Corresponding author:

Dennis Lal, PhD

Genomic Medicine Institute

Lerner Research Institute

Cleveland Clinic

Cleveland, OH 44195, US

Email: lald@ccf.org

Table of Contents

[1 Supplementary Data 3](#_Toc117769465)

[2 Example Analysis 6](#_Toc117769466)

[2.1 9q33.3q34.11 microdeletions 6](#_Toc117769467)

[2.2 Benign CNVs 11](#_Toc117769468)

[3 References 15](#_Toc117769469)

# 1 Supplementary Data

**Table S1. Data sources**

| **Category** | **Data** | **Description** | **Version / last acessed** | **Publication/ website** |
| --- | --- | --- | --- | --- |
| Chromosome | Coordinates of cytobands | Genomic coordinates and description of all cytobands of the human genome (hg19 and hg38). | October 21, 2021 | <http://hgdownload.soe.ucsc.edu/goldenPath/hg19/database/cytoBand.txt.gz>  <http://hgdownload.soe.ucsc.edu/goldenPath/hg38/database/cytoBand.txt.gz> |
| Genes and annotations | Protein coding genes | Gene list of all protein-coding genes. | October 21, 2021 | <https://www.genenames.org/> |
| Genes and annotations | RefSeq gene transcript coordinates | Genomic coordinates of all human transcripts (hg19 and hg38). | October 21, 2021, v 2020-08-17 | <http://hgdownload.soe.ucsc.edu/goldenPath/hg19/database/refGene.txt.gz>  <http://hgdownload.soe.ucsc.edu/goldenPath/hg38/database/refGene.txt.gz> |
| Genes and annotations | MANE select transcript (canonical transcripts) | High-quality representative transcript per protein-coding gene that is well-supported by experimental data and represents the biology of the gene. | October 21, 2021, v0.95 | <ftp://ftp.ncbi.nlm.nih.gov/refseq/MANE> |
| Genes and annotations | pLI/ leouf score | Predicted Constraint Metrics; pLI: Probability of loss of function intolerance; loeuf: Loss-of-function observed/expected upper bound fraction | August 11, 2021, v2.2.1 | [https://www.nature.com/articles/s41586-020-2308-7, https://www.nature.com/articles/nature19057](https://www.nature.com/articles/s41586-020-2308-7) |
| Genes and annotations | pHI/ pTS score | Haploinsufficiency and triplosensitivity scores for autosomal protein-coding genes; pTS: Probability of triplosensitivity; pHI: Probability of haploinsufficiency | August 20, 2021 | <https://www.medrxiv.org/content/10.1101/2021.01.26.21250098v1> |
| Genes and annotations | ClinGen gene-disease pairs | The ClinGen Gene Curation working group determines the clinical validity for gene-disease pairs using a standardized approach. | October 31, 2022 | <https://search.clinicalgenome.org/kb/gene-validity?page=1&size=25&search=> |
| Genes and annotations | %HI Decipher Haploinsufficiency Predictions | By DECIPHER updated predictions of haploinsufficiency as described by Huang et al., 2010. | October 21, 2021, v3 | <https://europepmc.org/article/MED/20976243,https://www.deciphergenomics.org/about/downloads/data> |
| Genes and annotations | ClinGen Dosage Sensitivity Curated Gene List | The ClinGen Dosage Sensitivity group collects evidence supporting/refuting the haploinsufficiency and triplosensitivity of genomic regions. | October 31, 2022 | <https://search.clinicalgenome.org/kb/downloads> |
| CNV data | ClinVar CNV data | ClinVar aggregates information about genomic variation and its relationship to human health. The data used here includes 52069 CNVs. | October 31, 2022 | <https://www.ncbi.nlm.nih.gov/clinvar/> |
| CNV data | UK Biobank CNV data | Allele count of copy number variants (n= 275,180) from 472,228 array-genotyped individuals from the UK Biobank. | June 26, 2019, v2 | <https://doi.org/10.1016/j.ajhg.2019.07.001> |
| CNV data | GnomAD SV data | SV/CNV allele frequency data from 14,891 genomes. | October 21, 2021, v2.1 | <https://www.nature.com/articles/s41586-020-2287-8> |
| Disease-associated genomic regions | CNV syndromes (DECIPHER) | Expert-curated microdeletion and microduplication syndromes involved in developmental disorders. | October 21, 2021 | <https://www.deciphergenomics.org/disorders/syndromes/list> |
| Dosage sensitive genomic regions | ClinGen Dosage Sensitivity Curated Region List | The ClinGen Dosage Sensitivity group collects evidence supporting/refuting the haploinsufficiency and triplosensitivity of genomic regions. | October 31, 2022 | <https://search.clinicalgenome.org/kb/downloads> |

**Fig. S1. Genomic Viewer of CNV-Clinviewer.** The genomic viewer enables the inspection of the uploaded CNVs and their genomic region alongside biomedical annotations and other pathogenic and general population CNV datasets. Users can interactively navigate by zooming in/out, moving, and selecting genomic regions of interest. **A)** The ideogram shows the selected genomic region. Users can select a genomic region of interest by Drag’n’Drop. **B)** In the gene track, all protein-coding genes are shown. Dosage-sensitive genes, based on a selection of different gene dosage sensitivity scores, are highlighted in orange for gene prioritization in context to the uploaded CNVs. **C)** Uploaded CNVs that intersect the selected region are visualized and can be interactively filtered based on uploaded phenotypic annotations, their assigned clinical significance, CNV type, and sample IDs. Deletions are visualized as red, duplications as blue bars. **D)** The ClinVar track has two tabs. The first tab (not shown) shows a visual summary of allele counts from pathogenic/ likely pathogenic CNVs. The second tab displays all CNVs from ClinVar in a plot grouped by their assigned clinical significance (pathogenic/ likely pathogenic vs. uncertain significance vs. benign/ likely benign) and can be filtered based on their type and clinical significance. Details about the ClinVar CNVs, such as their reported phenotypes or allele origin and the link to the ClinVar variant website, are shown in a table and can be downloaded for further analyses. **E)** Visualizations of summarized allele frequencies of large CNVs (>50kb) from the UK Biobank and **F)** gnomAD.

# 2 Example Analyses

## 2.1 9q33.3q34.11 microdeletions

To illustrate the utility of the CNV-ClinViewer with a proof-of-concept example, we used 9q33.3q34.11 microdeletions from the literature (n=14), along with the phenotypic status of phenotypes with high prevalence in the patient population (epilepsy, intellectual disability, finger dysplasia, facial dysmorphism)^1-4^. After uploading the data (Fig. S2), all CNVs are displayed in the table with their automatically assigned pathogenic (12/14 CNVs) or likely pathogenic (2/14 CNVs) classification (Fig. S3).


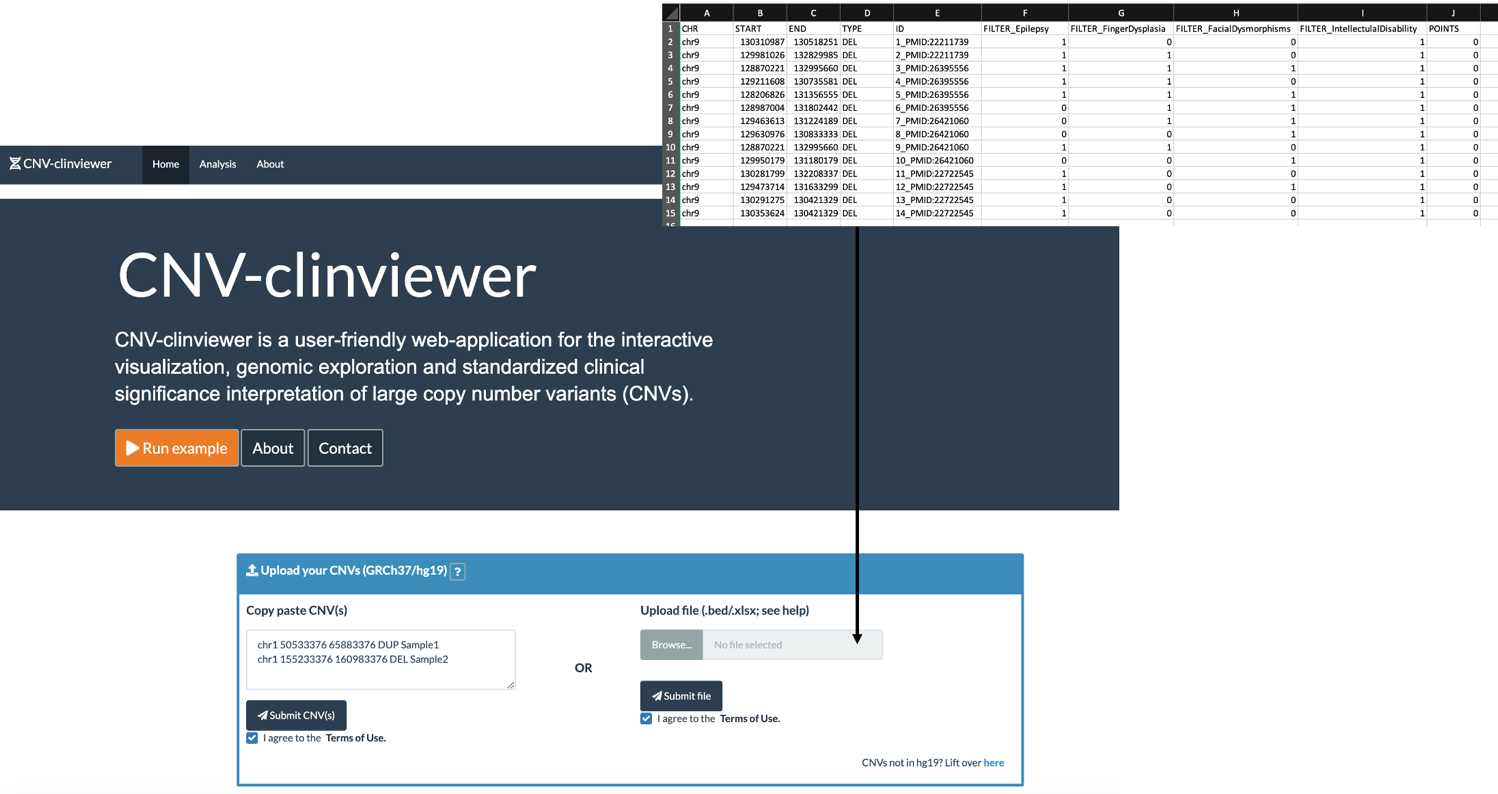


**Fig. S2: CNV data upload.** The data of 9q33.3q34.11 microdeletions (n=14) is uploaded as an Excel file with the required chromosome, start, end and type data, and additionally with sample IDs and binary phenotypes for filtering.


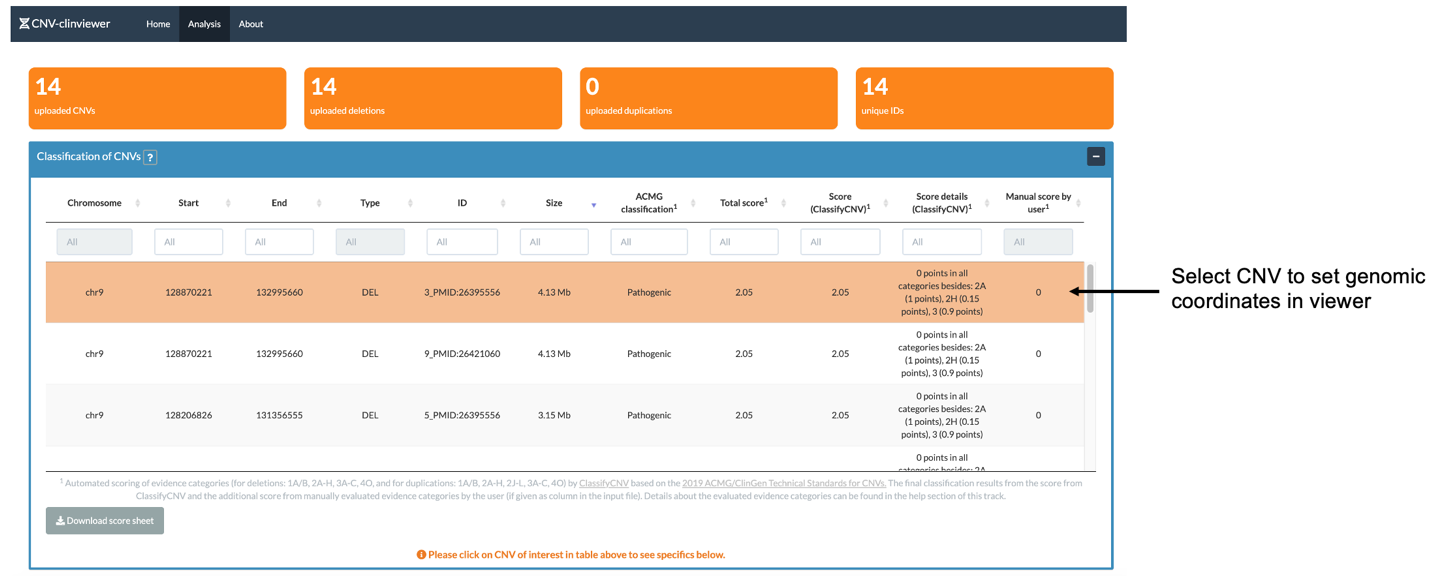


**Fig. S3: Table of uploaded and classified CNVs.** Upon upload of the data, the CNVs are classified by ClassifyCNV^5^ based on the 2019 ACMG/ClinGen guidelines and displayed in a table, including details about the scoring. Further information about the scoring of the evidence categories by ClassifyCNV can be retrieved in the help section of the panel by pressing the button next to the title of the panel. CNVs of interest can be selected in the table to download a comprehensive report and explore its genomic region and content in the genomic viewer.

From this table, we selected the largest CNV to set the genomic coordinates of interest for the genomic viewer and inspected the visualization of the uploaded CNVs and intersecting genes (Fig. S4). Here, one can observe that all CNVs overlap while their size is variable and that 18 genes in this region (e.g., *STXBP1* and *SPTAN1*) indicate dosage sensitivity by one or more of the integrated dosage sensitivity scores (highlighted in orange in the gene visualization).


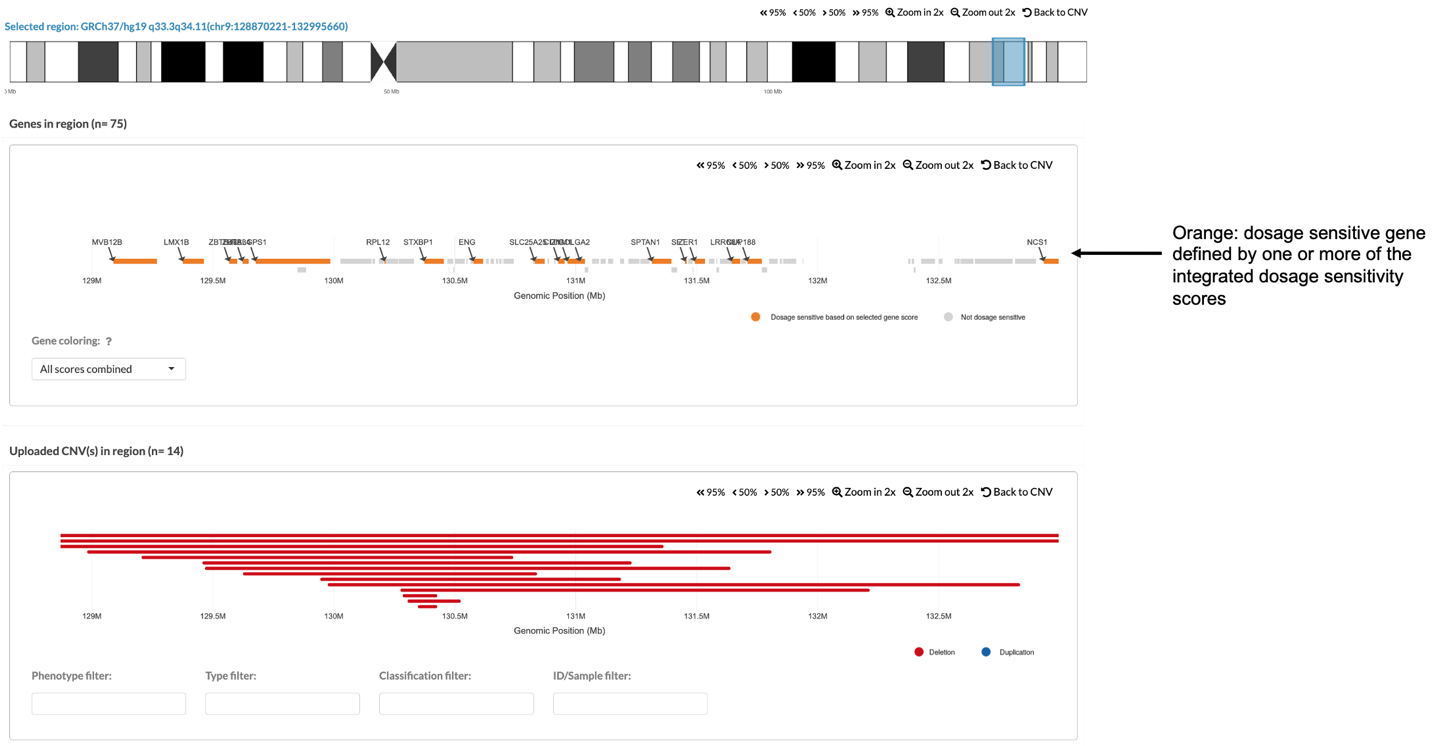


**Fig. S4: Genomic viewer with visualization of uploaded CNVs and intersecting genes.** Deletions are shown in red, and duplications are shown in blue (in this example there are only deletions). Dosage sensitive genes are displayed in orange to support the gene prioritization visually. The user can select from several dosage sensitive scores or can choose a combination of all.

To identify which gene(s) intersect the smallest region of overlap, we simply selected the plot area by Drag’n’Drop and identified the gene *STXBP1* (Fig. S5). By inspecting the individual dosage sensitivity scores in detail in the tooltip by hovering over the gene (Fig. S5) or in the gene table (Fig. S6), we found that all scores of *STXBP1* indicate dosage sensitivity, *e.g*., the haploinsufficiency score of 3 (= sufficient evidence) from ClinGen.


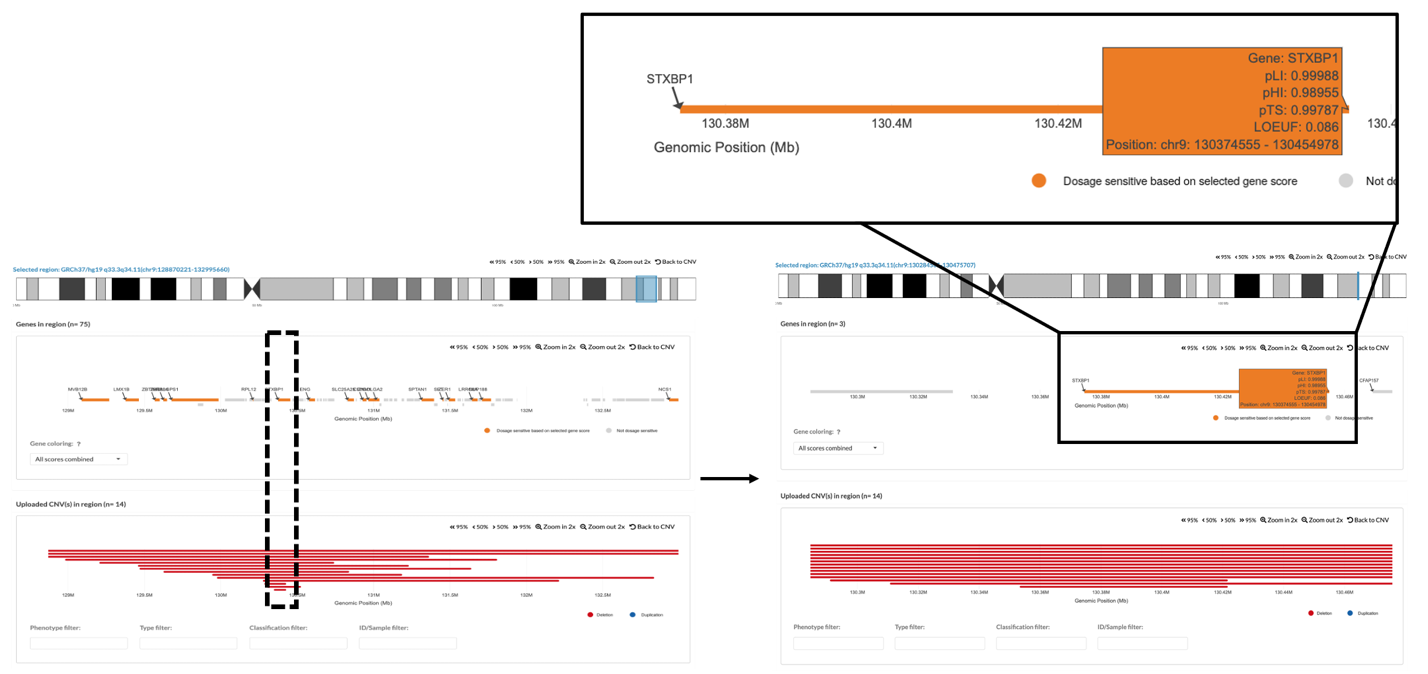


**Fig. S5: Identification of gene content in the smallest region of overlap of uploaded CNVs.** In order to zoom in the smallest region of overlap, the user can select the plot area by Drag’n’Drop. By hovering over genes and CNVs, the user can retrieve more information about those in a tooltip.

In addition, the table of intersecting gene-disease pairs from ClinGen lists *STXBP1* to be associated with developmental and epileptic encephalopathy (DEE) and links to the ClinGen online report (Fig. S6). Indeed, the gene is defined as one of the main causes of Ohtahara syndrome (OS), a devastating early onset DEE, and known as primary driver of 9q33.3q34.11 microdeletions^4^.


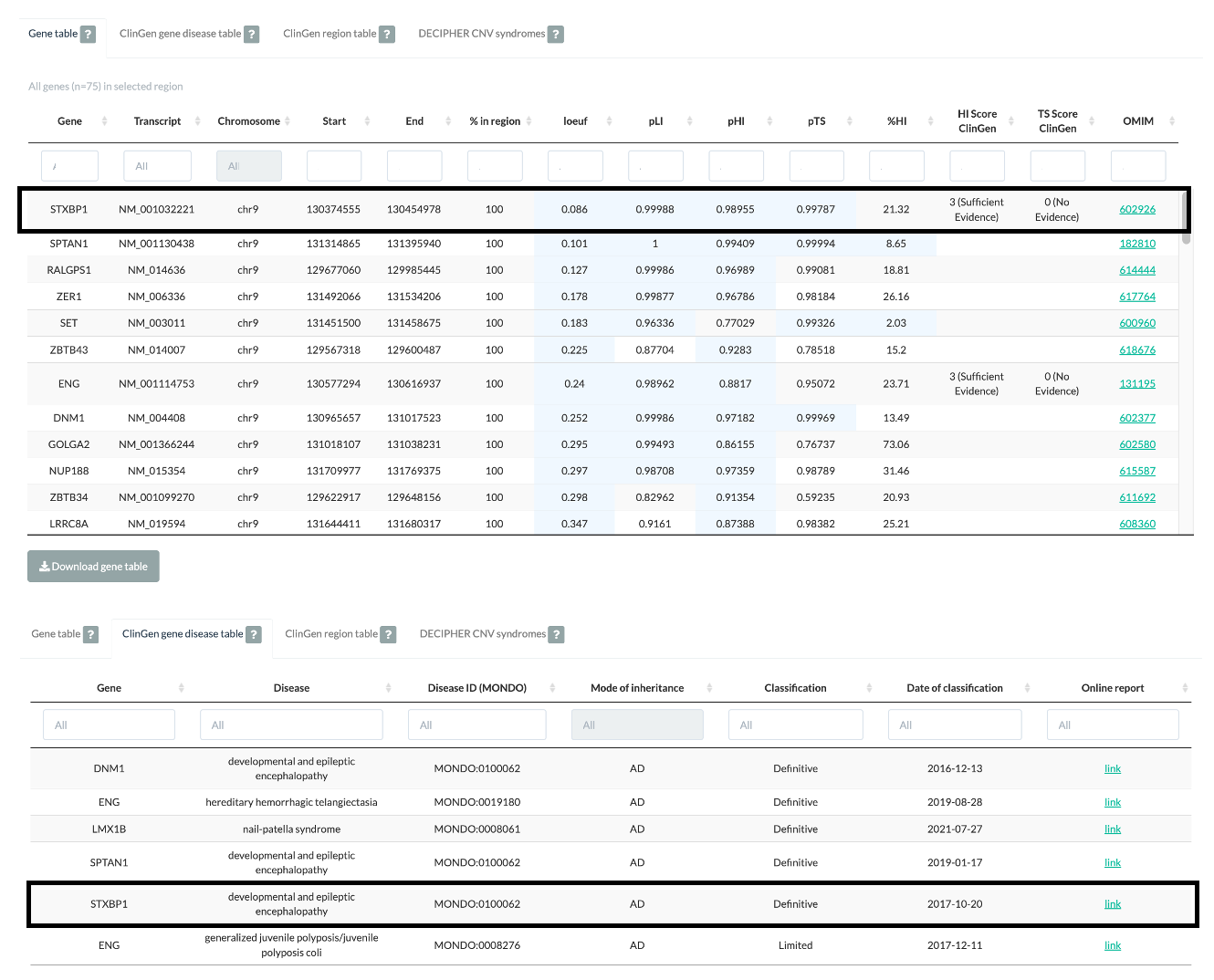


**Fig. S6: Gene table and ClinGen gene-disease association table.** The gene table includes all intersecting genes, annotated gene scores, and links to their OMIM website. In the ClinGen gene-disease association table, all genes associated with a disease in ClinGen, their disease ID (MONDO), their mode of inheritance, and link to the ClinGen online report, are displayed.

Zooming back to the larger region, we filtered the CNVs based on their phenotypes. We identified that patients with small microdeletions that only intersect *STXBP1* to have epilepsy and intellectual disability. At the same time, finger dysplasia and facial dysmorphism could only be seen in patients with larger CNVs, indicating more driver genes involved in their phenotypes (Fig. S7). The CNV-ClinViewer showed more genes in this region are associated with autosomal dominant diseases (*DNM1, ENG, SPTAN1* and *LMX1B*) (Fig. S6) which aligns with the conception that 9q34.11 genomic deletions involving *ENG*, *TOR1A*, *STXBP1*, and *SPTAN1* are revealing cis-genetic effects leading to complex phenotypes^1^.


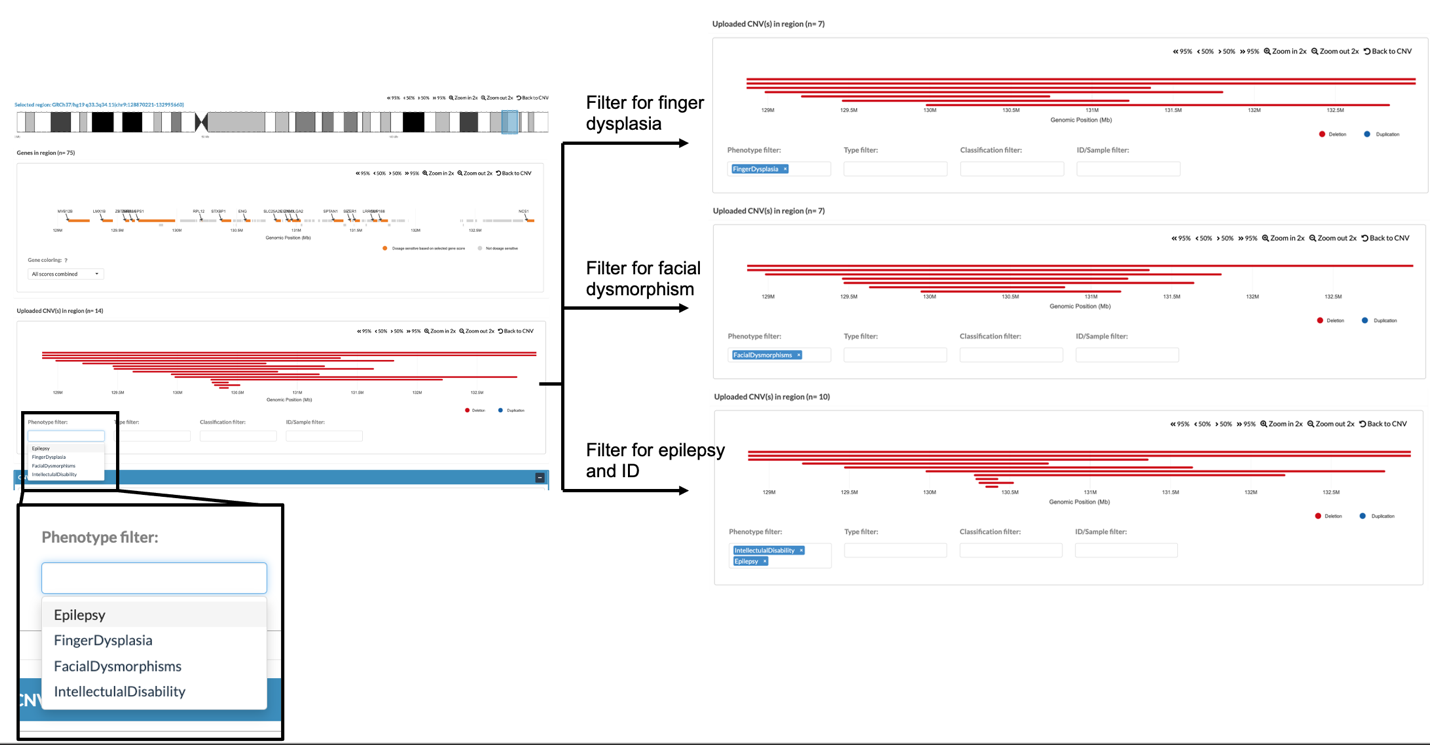


**Fig. S7: Dynamic filtering based on phenotypes of uploaded and visualized CNVs.** If binary phenotype information is provided in the input data, users can filter the CNVs based on those to perform genotype-phenotype analyses.

Besides the evaluation of the gene content, we could use the CNV-ClinViewer to identify overlap with 55 pathogenic/ likely pathogenic CNVs in ClinVar (15 deletions with 100% in the region), explore their phenotypes and get directed to the ClinVar variant websites for more information (Fig. S8).

**
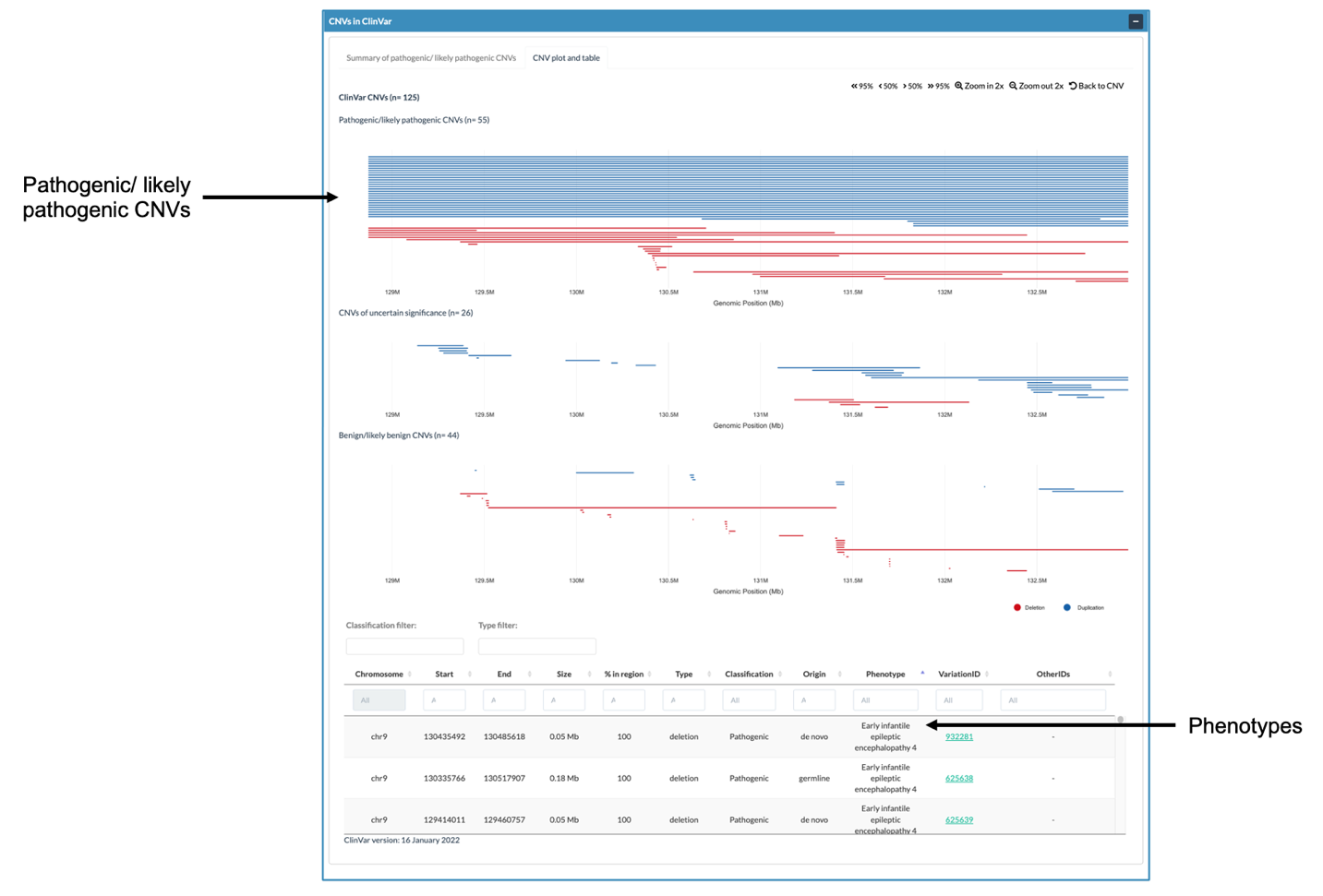
**

**Fig. S8: Identification of overlap with ClinVar CNVs**. The ClinVar track in the genomic viewer shows all intersecting CNVs from ClinVar, grouped by their clinical significance. The ClinVar CNVs can be filtered based on their classification and type. Detailed information about the phenotypes, origin, and links to their ClinVar reports can be found in a table below the visualization.

Overall, the CNV-ClinViewer successfully assisted the interpretation, exploration and analysis of the uploaded CNVs, and could replicate research findings that otherwise would require the usage of different databases and tools.

## 2.2 Benign 9q34.3q34.3 CNVs

To also illustrate the analysis of benign variants we selected 27 benign 9q34.3q34.3 CNVs from ClinVar. After uploading the CNVs, analogous to the prior example in 2.1, all CNVs are displayed in the table with their automatically assigned uncertain significance (15/27 CNVs) and benign (2/27 CNVs) classification (Fig. S9).

**
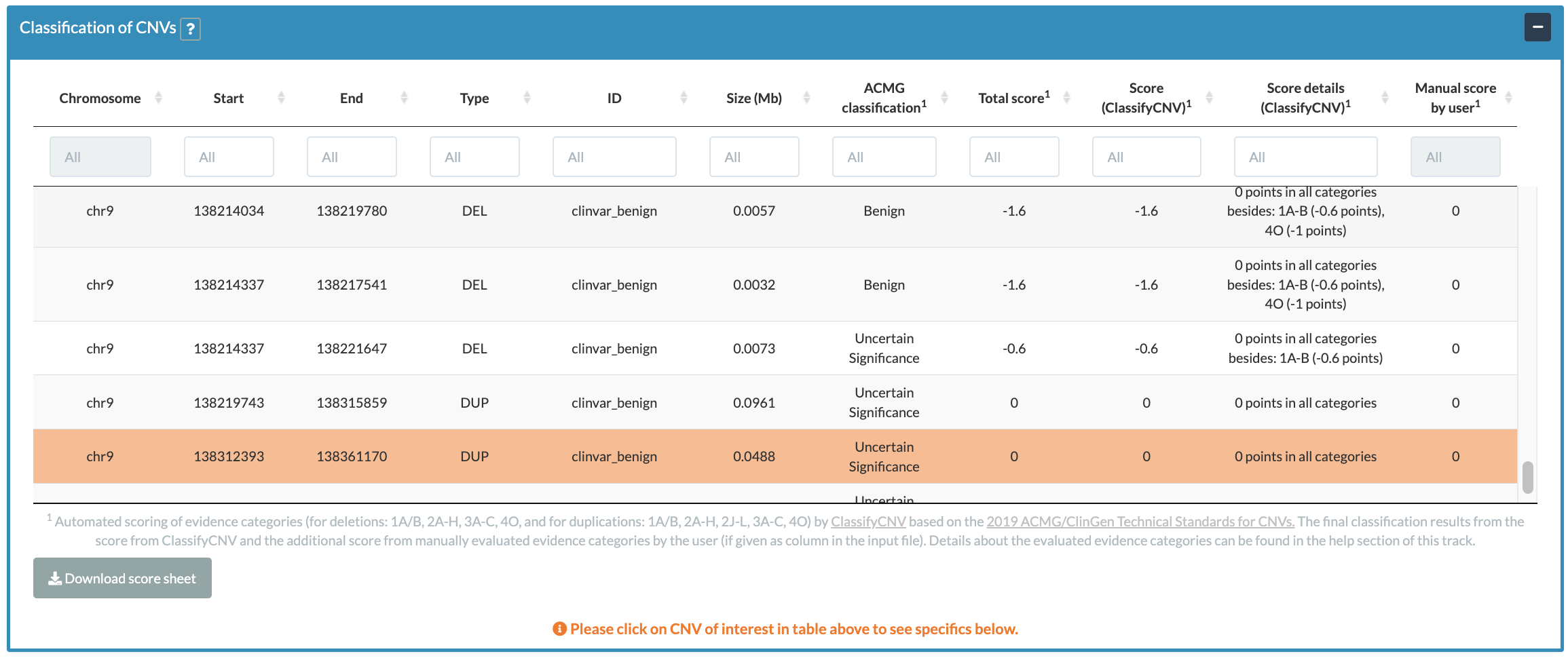
**

**Fig. S9: Table of uploaded and classified CNVs.** Upon upload of the data, the CNVs are classified by ClassifyCNV^5^ based on the 2019 ACMG/ClinGen guidelines and displayed in a table, including details about the scoring. CNVs of interest can be selected in the table to download a comprehensive report and explore its genomic region and content in the genomic viewer.

From this table, we selected a CNV to set the genomic coordinates for the genomic viewer and zoomed out to inspect the uploaded CNVs and intersecting genes (Fig. S10) in its surrounding region. Here, one can observe that the CNVs do not overlap with genes that indicate dosage sensitivity by one of the integrated dosage sensitivity scores.


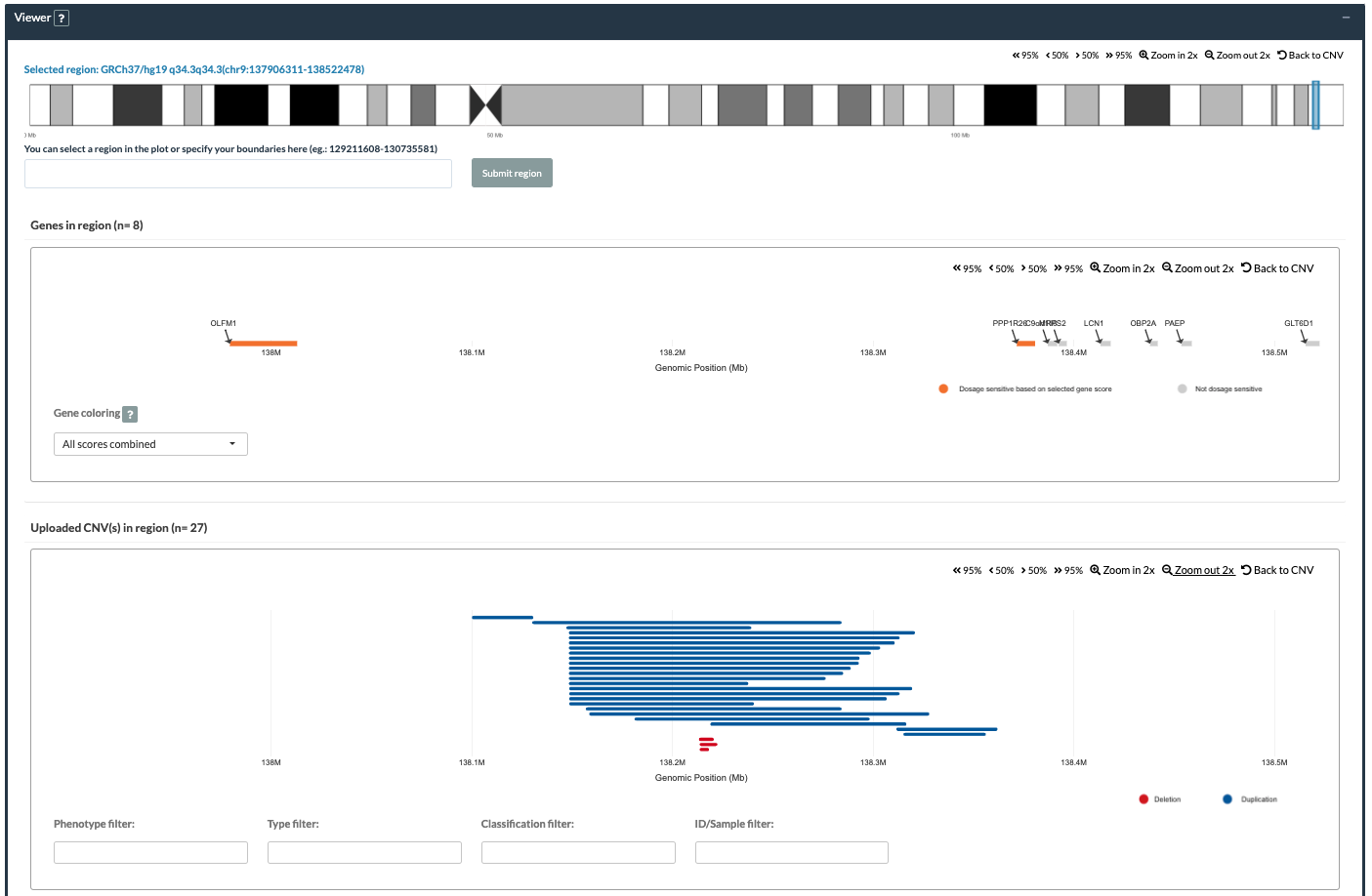


**Fig. S10:** **Genomic viewer with visualization of uploaded CNVs and intersecting genes.** Deletions are shown in red, and duplications are shown in blue. Dosage sensitive genes are displayed in orange.

Next, we explored the overlap of the benign variants with large (>50kb) population CNVs from the UK-Biobank and gnomAD. Here, we detected an increased allele frequency (Fig. S11) and highly overlapping duplications and deletions (Fig. S12) with our uploaded CNVs.

Overall, the CNV-ClinViewer successfully replicated the classification from ClinVar. Although the majority of the CNVs were not classified as benign by the automated classification algorithm, the additional visual exploration of the uploaded CNVs alongside integrated CNV datasets increased the confidence that the CNVs are benign.


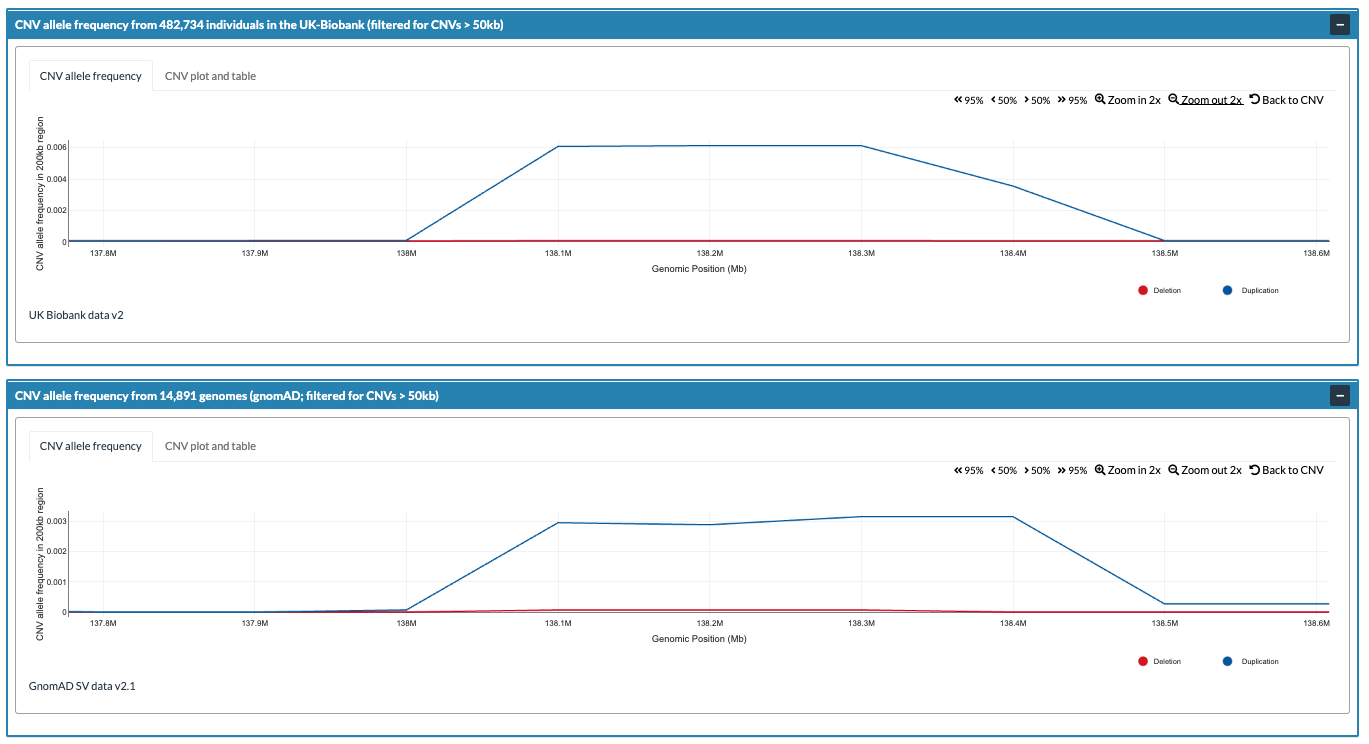


**Fig. S11: Visualization of summarized allele frequency of large CNVs in the UK-Biobank and gnomAD.** The allele frequency of deletions are shown in red, and the allele frequency of duplications are shown in blue.


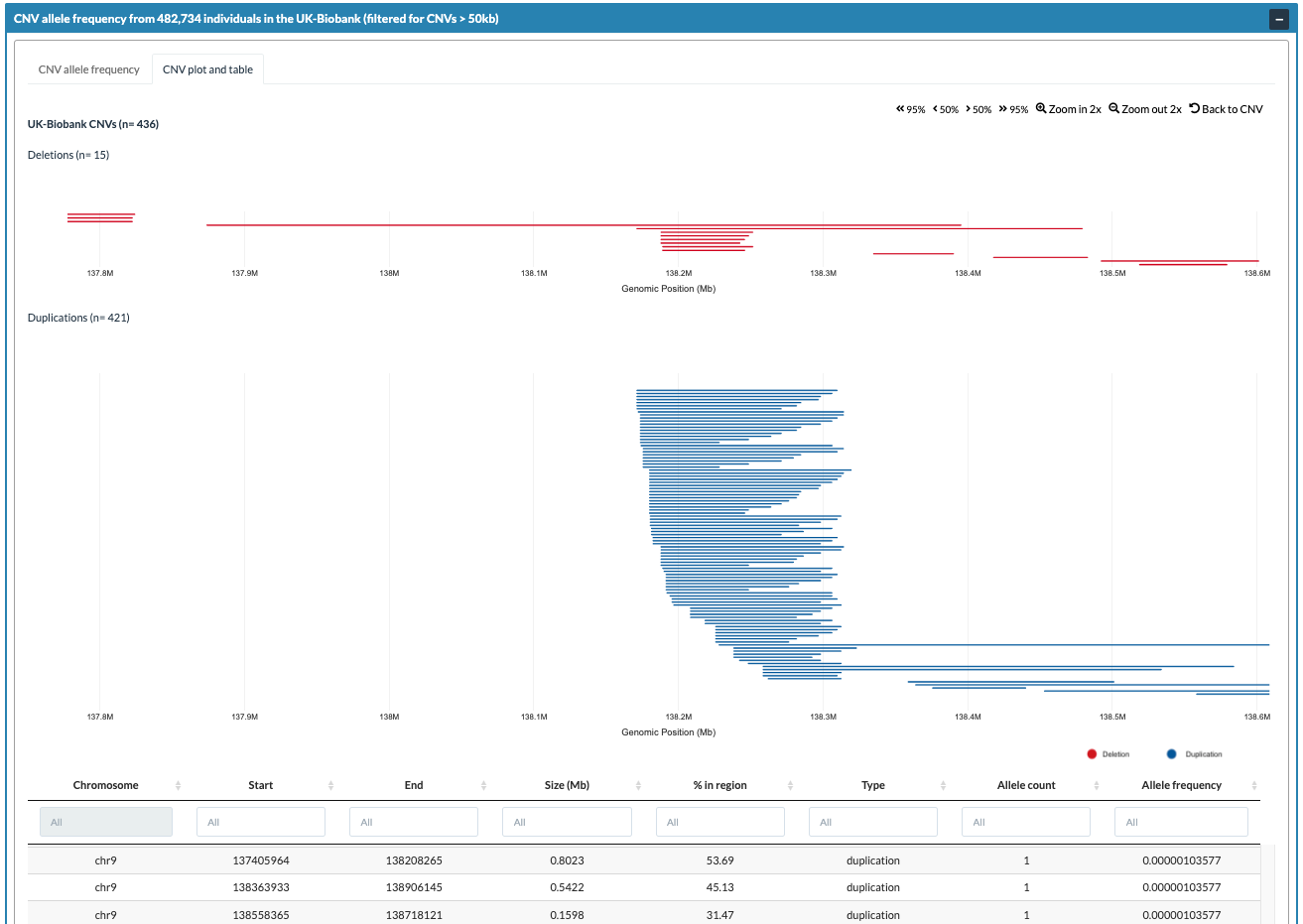


**Fig. S12: Visualization of large CNVs (>50 kb) in the UK-Biobank.** Deletions are shown in red, and duplications are shown in blue.

# 3 References

1. Campbell IM, Yatsenko SA, Hixson P, et al. Novel 9q34.11 gene deletions encompassing combinations of four Mendelian disease genes: STXBP1, SPTAN1, ENG, and TOR1A. *Genet Med*. 2012;14(10):868-876. doi:10.1038/gim.2012.65

2. Ehret JK, Engels H, Cremer K, et al. Microdeletions in 9q33.3-q34.11 in five patients with intellectual disability, microcephaly, and seizures of incomplete penetrance: is STXBP1 not the only causative gene? *Mol Cytogenet*. 2015;8:72. doi:10.1186/s13039-015-0178-8

3. Nambot S, Masurel A, El Chehadeh S, et al. 9q33.3q34.11 microdeletion: new contiguous gene syndrome encompassing STXBP1, LMX1B and ENG genes assessed using reverse phenotyping. *Eur J Hum Genet*. 2016;24(6):830-837. doi:10.1038/ejhg.2015.202

4. Saitsu H, Kato M, Shimono M, et al. Association of genomic deletions in the STXBP1 gene with Ohtahara syndrome. *Clin Genet*. 2012;81(4):399-402. doi:10.1111/j.1399-0004.2011.01733.

**5.** Gurbich TA, Ilinsky VV. ClassifyCNV: a tool for clinical annotation of copy-number variants. *Sci Rep*. 2020;10(1):20375. doi:10.1038/s41598-020-76425-3
